# Digital Biomarkers of Physical Frailty and Frailty Phenotypes Using Sensor-Based Physical Activity and Machine Learning

**DOI:** 10.3390/s21165289

**Published:** 2021-08-05

**Authors:** Catherine Park, Ramkinker Mishra, Jonathan Golledge, Bijan Najafi

**Affiliations:** 1 Interdisciplinary Consortium on Advanced Motion Performance (iCAMP), Michael E. DeBakey Department of Surgery, Baylor College of Medicine, Houston, TX 77030, USA; catherine.park@bcm.edu (C.P.); ram.mishra@bcm.edu (R.M.); 2 Queensland Research Centre for Peripheral Vascular Disease, Australian Institute of Tropical Medicine, College of Medicine and Dentistry, James Cook University, Townsville, QLD 4811, Australia; Jonathan.golledge@jcu.edu.au; 3 The Department of Vascular and Endovascular Surgery, The Townsville University Hospital, Townsville, QLD 4814, Australia

**Keywords:** digital biomarkers, physical frailty, frailty phenotype, machine learning, wearable, remote patient monitoring, physical activity, older adults, digital health, digital twins

## Abstract

Remote monitoring of physical frailty is important to personalize care for slowing down the frailty process and/or for the healthy recovery of older adults following acute or chronic stressors. Taking the Fried frailty criteria as a reference to determine physical frailty and frailty phenotypes (slowness, weakness, exhaustion, inactivity), this study aimed to explore the benefit of machine learning to determine the least number of digital biomarkers of physical frailty measurable from a pendant sensor during activities of daily living. Two hundred and fifty-nine older adults were classified into robust or pre-frail/frail groups based on the physical frailty assessments by the Fried frailty criteria. All participants wore a pendant sensor at the sternum level for 48 h. Of seventeen sensor-derived features extracted from a pendant sensor, fourteen significant features were used for machine learning based on logistic regression modeling and a recursive feature elimination technique incorporating bootstrapping. The combination of percentage time standing, percentage time walking, walking cadence, and longest walking bout were identified as optimal digital biomarkers of physical frailty and frailty phenotypes. These findings suggest that a combination of sensor-measured exhaustion, inactivity, and speed have potential to screen and monitor people for physical frailty and frailty phenotypes.

## 1. Introduction

Physical frailty is a clinical syndrome characterized by decreased physiological reserve and function [1]. It is prevalent in older adults [2,3] and is typically a chronic and progressive health condition related to the aging process [4,5,6]. Physical frailty increases the risk of adverse health outcomes, such as falls, disability, hospital admission, and mortality [3,7,8]. It is also associated with increased requirement for medical care [9] and higher health care costs [10]. Effective tests to identify older adults who are at risk or currently have physical frailty may be valuable [11], because the condition can be potentially ameliorated or reversed if treated early enough [12,13,14].

Of more than 60 frailty assessment tools in clinical use [15], the two most common tools are the frailty phenotype and frailty index. The frailty phenotype defines physical frailty as a physical condition by assessing the five phenotypes, slowness, weakness, exhaustion, weight loss, and inactivity (low physical activity) [2]. Slowness and weakness are assessed by a 4.57 m walking test and a grip strength test, respectively, and exhaustion, weight loss, and inactivity are assessed by an individual’s responses to a questionnaire. The health professional identifies pre-frail/frail when an individual presents one or more of the five phenotypes. The frailty index defines physical frailty as an accumulation of health deficits (e.g., signs, symptoms, disabilities, laboratory abnormalities, diseases, etc.) based on an individual’s responses to a questionnaire asking about symptoms and other health conditions [16]. The health professional calculates the frailty index as a ratio between the number of identified deficits and the number assessed.

Although the frailty phenotype and frailty index have been extensively validated and widely used for physical frailty assessments in research and clinical practice, these two common tools are resource-intensive, requiring equipment, space, and trained health professionals [17], impractical in busy clinical settings [18], insensitive to subtle physiological changes [19], and not suitable for remote assessments. Additionally, both tools may not correctly identify physical frailty due to reliance on subjective patient-reported outcomes. For these and other reasons, there has been growing interest in developing wearable sensors as an alternative to existing frailty assessment tools.

Multiple studies have reported that sensors worn during daily living activities can identify physical frailty [20,21,22,23,24,25,26,27,28,29]. Past studies have demonstrated that a 20 s repetitive elbow flexion and extension task with a wrist sensor could identify physical frailty in community-dwelling older adults [20] and bed-bound older adults [21]. Recently, we demonstrated that a five-times sit-to-stand test performed while wearing sensors identified physical frailty in older adults [22] and that a machine learning method identified three optimal sensor-derived features representing slowness, weakness, and exhaustion [23]. The methodologies proposed in these studies, however, required supervised administration of frailty examination (e.g., 20 s repetitive elbow flexion and extension test, 5-times sit-to-stand test, gait, etc.), which may not be suitable for tracking frailty and its phenotypes during activities of daily living. A few recent studies suggested sensor-based in-home assessment of frailty via measuring activity levels (e.g., sedentary behavior, light and moderate–vigorous physical activity) [25,26]. Schwenk et al. have reported that posture parameters (e.g., standing, sitting, and lying) and gait parameters (e.g., speed, velocity, and cadence) measured using sensors worn during in-home assessments discriminated the frailty statuses [27]. However, these studies often included small sample sizes and explored limited features which may not represent different phenotypes of frailty including slowness, weakness, and exhaustion.

Notably, two studies have demonstrated the feasibility, acceptability, and reliability of identifying the frailty statuses using a single sensor worn by older adults during daily home monitoring [28,29]. Parvaneh et al. have reported that daily postural transitions (e.g., stand-to-walk, walk-to-stand, and total number of transitions) and postural characteristics (e.g., ratio of cautious sitting) identified pre-frailty [28]. Razjouyan et al. have reported that activity levels (e.g., sedentary behavior, light and moderate–vigorous physical activity) and activity characteristics (standing, longest walking bout, longest stepping bout) identified the frailty statuses [29]. While these two studies report that physical frailty can be identified using a single sensor worn during daily home monitoring, extracting and analyzing a large amount of sensor-derived data is both complex and infeasible for all applications. Furthermore, the assessed physical activity features were limited and may not represent all phenotypes of frailty, such as slowness, weakness, and exhaustion. Furthermore, in prior studies, participants with cognitive impairment or major chronic illness were often excluded, which may not represent the general frailty population who could benefit from remote patient monitoring.

The proposed study aims to address the gaps described above by exploring a machine learning-driven methodology to identify the lowest number of digital biomarkers of physical frailty measurable using a pendant sensor. In addition, we explored the physical activity features that may best represent individual frailty phenotypes including slowness, weakness, exhaustion, and inactivity. To achieve these aims, we collected a relatively large number of older adults, including both community-dwelling older adults and an outpatient geriatric population, with various comorbidities, to better represent older adults who may benefit from frailty tracking via remote patient monitoring. We hypothesized that combining a machine learning technique with frailty modeling would determine the optimal features needed for identifying physical frailty, slowness, weakness, exhaustion, and inactivity.

## 2. Materials and Methods

### 2.1. Participants and Experimental Protocols

Participants in this study were recruited from the Baylor College of Medicine and University of Arizona for the purpose of different observational or clinical trials focused on older adults. Eligible individuals were aged 65 years or older and able to independently walk a distance of at least 10 m with or without walking assistance devices (e.g., walker or cane). Potential participants were excluded if they: (1) were unable to walk a distance of 10 m, or unable to stand still without moving their feet; (2) had a foot problem that may affect their ability to walk, such as active foot ulcers or infection, major foot deformity, or major amputation; (3) had a medical or psychiatric condition deemed to be a contraindication to ambulation (e.g., recent stroke, unstable from pulmonary embolus, unstable angina, severe hemodynamic instability, delirium, severe cognitive impairment, and severe depression) affecting mobility; (4) were involved in an ongoing therapy such as physical therapy or any other exercise intervention, occupational therapy, chemotherapy, mental health, and social work services, or (5) were not willing to participate. Additional exclusion criteria specific to this study were lack of availability of data from physical activity monitoring for a period of at least 48 h and lack of availability of an assessment of the Fried frailty criteria. For the eligible subjects selected from the prospective studies, only data from baseline with no intervention were included in final data analysis.

The study protocol was approved by the local Institutional Review Boards at Baylor College of Medicine (Protocols: H-43917, H-41417, H-40765, H-38347, H-37792) and University of Arizona (Protocol: 12-0659-01). All participants reviewed and signed a consent form prior to the study.

Demographic information collected from the participants consisted of age, gender, height, weight, and body mass index (BMI). The Fried frailty phenotype, which assesses physical frailty ranging from 0 to 5 based on the 5 phenotypes (slowness, weakness, exhaustion, weight loss, and inactivity) [2], was used to classify participants into a robust group (RG) or a pre-frail/frail group (FG). The RG had no phenotypes, while the FG had 1 or more of the 5 phenotypes.

**Figure 1:**
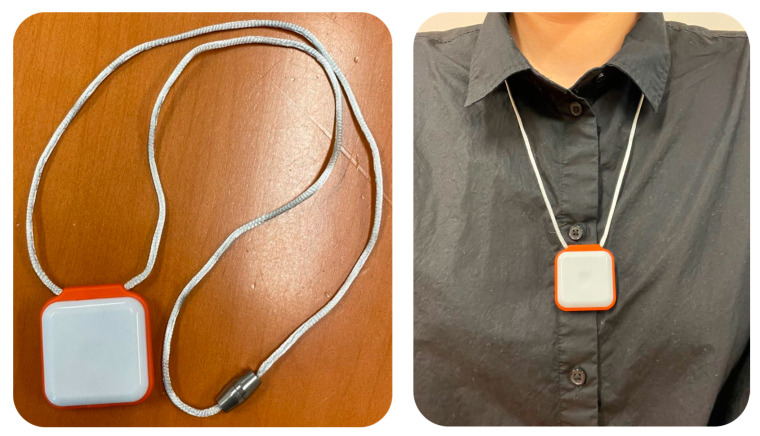
The PAMSys wearable sensor and its placement.

Participants wore a pendant sensor (PAMSys™, BioSensics, Newton, MA, USA) at the sternum level for two consecutive days (48 h), as shown in Figure 1. The 48 h duration for data collection was determined based on the results of our previous studies [29,30]. The PAMSys™ includes a tri-axial accelerometer and gyroscope, and is small (3.5 cm (W) × 3.5 cm (H) × 1.5 cm (D)) and light in weight (24 g) (BioSensics, Newton, MA, USA). The manufacturer’s specifications indicate that the PAMSys™ uses advanced signal processing algorithms and novel biomechanical models of human motion to continuously record posture, gait, and physical activity data at a rate of 50 Hz. The PAMSys™ can run for 200 h without charging. It has built-in memory to save data.

### 2.2. Sensor-Derived Features

All sensor-derived features are downloaded from the sensor’s built-in memory using PAMWare™ (BioSensics, Newton, MA, USA). Based on previous studies [25,26,27,28,29,30], we used 12 sensor-derived features. Table 1 describes the 12 features and associated frailty phenotypes. Each feature was computed per 24 h and each feature for 48 h was averaged [29].

### 2.3. Data Analysis and Optimal Feature Selection Using Machine Learning

All statistical analyses were conducted using SPSS (IBM Corp., Armonk, NY, USA). Outcome measures were the participants’ demographics and frailty phenotypes and the 16 sensor-derived features.

The Shapiro–Wilk test was used to assess the distribution of the continuous data. A one-way analysis of variance (ANOVA) was conducted for normally distributed demographics, and a Mann–Whitney U test was conducted for non-normally distributed demographics. A chi-square test was used to test categorical data (i.e., gender). For the 12 sensor-derived features, linear mixed model for normally distributed data and generalized estimating equations for non-normally distributed data were conducted to assess the main effect of the groups, adjusting for BMI since it differed statistically between the RG and FG. Least significant difference method was used for multiple pairwise comparisons. Effect sizes were computed using Cohen’s d for the 12 sensor-derived features [31]. To assess whether significant features among the 12 sensor-derived features could identify physical frailty and frailty phenotypes, five models (physical frailty, slowness, weakness, exhaustion, and inactivity models) were built using binary logistic regression. The presence and absence of physical frailty, slowness phenotype, weakness phenotype, exhaustion phenotype, and inactivity phenotype were used as the dependent variable for the frailty, slowness, weakness, exhaustion, and inactivity models. The independent variables were significant sensor-derived features determined by the linear mixed model or generalized estimating equations to be significantly associated with physical frailty. An area-under-curve (AUC) was calculated to evaluate model performance [32]. For all statistical analyses, the level of significance was set at the 2-sided *p* < 0.05.

To determine the lowest number of sensor-derived features required to best identify physical frailty, optimal feature selection using machine learning with logistic regression modeling was used. The 11 significant sensor-derived features were used as the independent variables for the logistic regression modeling. Frailty status (0 (robust) or 1 (frail)) was used as the dependent variable. The machine learning algorithm was based on a bootstrapping technique and a recursive feature elimination technique [23]. The bootstrapping technique was used to generalize logistic regression modeling [33,34]. As recommended in [34], 2000 bootstrap iterations were used to optimize the robustness of logistic regression modeling in line with the number of participants (*n* = 259). The recursive feature elimination technique was used to rank the most effective features for optimal logistic regression modeling performance [21].

The machine learning algorithm had six steps:

(1) In total, 2000 pairs of training and validation datasets were created from the participants’ data (*n* = 259) using the bootstrapping technique. To avoid the possible misidentifications of physical frailty and frailty phenotypes due to the difference in the number of samples between groups (i.e., RG (*n* = 73) and FG (*n* = 186)), the FG was randomly subsampled by 73 at creating each pair of training and validation datasets.
(2) Logistic regression models were built at each recursive loop using the 2000 pairs of training datasets. In each recursive loop, the number of logistic regression models and the number of significant features considered were the same (e.g., the first recursive loop created 14 logistic regression models, and 14 logistic regression models decreased by 1 after each recursive loop).
(3) At each recursive loop, the AUC of each model was calculated.
(4) The AUC values were averaged across 2000 iterations for each model.
(5) A feature with the lowest AUC value was removed.
(6) Steps 2–5 repeated until only one feature remained (steps 2–5 corresponded to one recursive loop, and each recursive loop ran 2000 iterations).

The machine learning algorithm ran 130,000 iterations in total:

(1) $$130,000\;\mathrm{iterations}\;=2000\;\mathrm{pairs}\;\mathrm{of}\;\mathrm{resampling}\;\times \frac{n\left(a+l\right)}{2}$$

where *n*, *a*, and *l* indicate a number of terms (i.e., 10 = 11 (the number of significant features) − 1), 11 (i.e., the number of significant features considered in the first recursive loop), and 2 (i.e., the number of significant features considered in the last recursive loop).

AUC, sensitivity, specificity, accuracy, positive predictive value (PPV), negative predictive value (NPV), mean, and 95% confidence interval (CI) were computed from the 2000 pairs of training datasets to evaluate the performance of the logistic regression models. Using the 2000 pairs of validation datasets, the performance of a logistic regression model with optimal features was also evaluated. The PPV and NPV were computed as:

(2) $$\mathrm{PPV}\;=\frac{\mathrm{true}\;\mathrm{positive}}{\mathrm{true}\;\mathrm{positive}+\mathrm{false}\;\mathrm{positive}}\times 100\;\left(\%\right)$$

(3) $$\mathrm{NPV}\;=\frac{\mathrm{true}\;\mathrm{negative}}{\mathrm{true}\;\mathrm{negative}+\mathrm{false}\;\mathrm{negative}}\times 100\;\left(\%\right)$$

## 3. Results

Two hundred and fifty-nine older adults (Age = 76.0 ± 9.8, BMI = 27.8 ± 8.2, 64.9% female, 71.8% frail) satisfied the inclusion and exclusion criteria for this study. Table 2 reports the results of participants’ demographics and frailty phenotypes for the RG and FG. Statistical analysis of participants’ demographics showed that BMI significantly differed between the RG and FG. However, age, gender, height, and weight were not significantly different between the RG and FG. In the FG, 69%, 64%, 30%, 9%, and 40% of participants had slowness, weakness, exhaustion, weight loss, and inactivity, respectively.

### 3.1. Significant Sensor-Derived Features

Table 3 reports the descriptive statistics and statistical results of the RG and FG for the 16 sensor-derived features. Among 12 sensor-derived features, 11 sensor-derived features significantly differed between the groups.

Compared to the RG, the FG had significantly slower walking cadence, higher number of stand-to-sit, longer duration of stand-to-sit, higher number of sit-to-stand, and longer duration of sit-to-stand, which are features of slowness/weakness. The FG had significantly shorter longest walking bout and walking steps per episode, which are features of exhaustion. The FG had significantly less walking steps, higher % of sitting, less % of standing, and less % of walking, which are features of inactivity. The Cohen’s d effect size was large (0.8–1.29) for two sensor-derived features (% of standing and % of walking), medium (0.5–0.79) for four sensor-derived features (walking cadence, duration of sit-to-stand, longest walking bout, and walking steps), and small (0.2–0.49) for five sensor-derived features (number of stand-to-sit, duration of stand-to-sit, number of sit-to-stand, walking steps per episode, and % of sitting).

Table 4 reports the results of binary logistic modeling and AUC for the physical frailty, slowness, weakness, exhaustion, and inactivity models. The AUCs of the physical frailty, slowness, weakness, exhaustion, and inactivity model were 0.80, 0.74, 0.71, 0.60, and 0.72, respectively. The AUC of the physical frailty model was within an excellent range (0.8 ≤ AUC < 0.9), the AUCs of the slowness, weakness, and inactivity models were within an acceptable range (0.7 ≤ AUC < 0.8), and the AUC of the exhaustion model was within a fair range (0.6 ≤ AUC < 0.7).

### 3.2. Optimal Feature Selection and Evaluation

**Figure 2:**
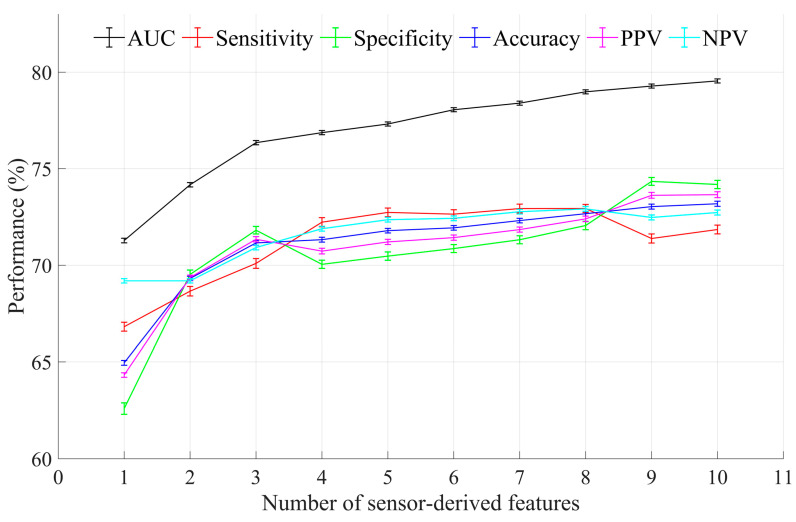
Results of optimal feature selection using machine learning. Error bars indicate 95% confidence intervals. AUC, PPV, and NPV indicate area under the receiver operating characteristic curve, positive predictive value, and negative predictive value, respectively.

Table 5 reports the ranking of the 11 sensor-derived features used for optimal feature selection with machine learning. Figure 2 shows AUC, sensitivity, specificity, and accuracy as a function of the ranked features. A logistic regression model with all 11 sensor-derived features had an AUC of 79.5% (95% CI = 79.4–79.7), a sensitivity of 71.8% (95% CI = 71.6–72.1), a specificity of 74.2% (95% CI = 74.0–74.4), an accuracy of 73.2% (95% CI = 73.1–73.3), a PPV of 73.7% (95% CI = 73.5–73.8), and an NPV of 72.7% (95% CI = 72.6–72.9). For the slowness/weakness, exhaustion, and inactivity phenotypes and an acceptable AUC of 0.7 to 0.8 [35], % of standing, % of walking, walking cadence, and longest walking bout were identified as the optimal features. A logistic regression model with these features had an AUC of 76.9% (95% CI = 76.7–77.0), a sensitivity of 72.2% (95% CI = 72.0–72.5), a specificity of 70.0% (95% CI = 69.8–70.3), an accuracy of 71.3% (95% CI = 71.2–71.4), a PPV of 70.7% (95% CI = 70.6–70.9), and an NPV of 71.9% (95% CI = 71.8–72.0), as shown in Figure 2. The equation of the logistic regression model with the optimal features was:

(4) $$\ln\left(\frac{p\left(X\right)}{1-p\left(X\right)}\right)=\beta_{0}+\beta_{1}X_{1}+\beta_{2}X_{2}+\beta_{3}X_{3}+\beta_{4}X_{4}$$

where p(X) is the probability of robust or frail ranging between 0 and 1, X_1_, X_2_, X_3_, and X_4_ indicate % of standing, % of walking, walking cadence, longest walking bout, respectively. β_0_ is intercept (6.1883), and β_1_, β_2_, β_3_, and β4 are constant coefficients (β_1_ = −0.0885, β_2_ = −0.1405, β_3_ = −0.0003, and β_4_ = −0.0341), respectively.

Table 6 reports the model validations computed by applying the validation datasets to the logistic regression model with the optimal features.

## 4. Discussion

This study examined the association between the physical activity features measurable remotely from a pendant sensor and physical frailty and its phenotypes based on machine learning and modeling of physical frailty and physical phenotypes. While various tools for identifying physical frailty have been used in clinical and research settings (see [17,36] for review), they are mostly based on subjective patient-reported outcomes using questionnaires, and/or have to be administered face-to-face. The literature has stressed the importance of routine physical frailty assessments [2,37], since physical frailty can be reversible if detected and treated in a timely manner [13]. Therefore, sensor-based frailty assessments have been proposed and assessed. While reliable functional tests such as balance, gait, five-times sit-to-stand, and timed up-and-go recorded from wearable sensors have been reported to be associated with functional performance and physical frailty [22,23,24,27,28,38,39], the test results were based on supervised assessments of motor performance that are unsuitable for the remote monitoring of physical frailty under unsupervised conditions. Instead, the advantage of using unsupervised daily physical activity monitoring to determine frailty is its practicality for remote monitoring and tracking changes in frailty statuses over time. Wearable-based daily activity monitoring has been reported to accurately identify physical frailty [25,26,27,28,29]. The use of wearable-based daily activity monitoring as a remote patient monitoring tool may assist in the adoption of routine frailty screening in the clinical setting for the purpose of personalized care and optimized care decision planning, because the technology does not require dedicated space, clinical personnel, and the specific motor skills needed for functional tests. Although a few studies have used a single wearable sensor [27,28,29], this study is the first to identify the optimal sensor-derived clinically meaningful features required for identifying physical frailty and frailty phenotypes.

Consistent with previous studies, our results showed that posture, gait, and physical activity parameters measured by a single wearable sensor were associated with physical frailty [24,25,26,27,28,29]. Specifically, the 11 sensor-derived features (i.e., walking cadence, number of stand-to-sit, duration of stand-to-sit, number of sit-to-stand, duration of sit-to-stand, longest walking bout, walking steps per episode, walking steps, % of sitting, % of standing, and % of walking) differed significantly between the RG and FG. While five sensor-derived features (number of stand-to-sit, duration of stand-to-sit, number of sit-to-stand, walking steps per episode, and % of sitting) had a small effect size, six other features had a medium or large effect size. The five sensor-derived features with a small effect size may not be sensitive enough to distinguish physical frailty. We also attribute the small effect size to an imbalanced number of participants between the RG (*n* = 73) and FG (*n* = 186). Five models using the 11 significant sensor-derived features and binary logistic regression identified physical frailty and frailty phenotypes (slowness, weakness, exhaustion, and inactivity). The results of our logistic regression modeling using the 11 significant sensor-derived features and binary logistic regression showed a fair to excellent AUC [40]. Specifically, the physical frailty model had an excellent AUC (≥0.8), the slowness, weakness, and inactivity models had acceptable AUCs (≥0.7), and the exhaustion model had a fair AUC (≥0.6).

Optimal feature selection using machine learning determined the optimal features (i.e., optimal digital biomarkers) as % of standing (indicator of inactivity), % of walking (indicator of inactivity), walking cadence (indicator of slowness/weakness), and longest walking bout (indicator of exhaustion). These four optimal features had a medium or large effect size. Using 2000 pairs of training and validation datasets with a subsampling technique, the performance of logistic regression modeling with the optimal sensor-derived features showed an acceptable AUC (76.9%), sensitivity (72.2%), specificity (70.0%), accuracy (71.3%), PPV (70.7%), and NPV (71.9%). Our results also indicated that the physical frailty model with the four optimal features had a similar performance level of AUC, sensitivity, specificity, accuracy, PPV, and NPV compared to the physical frailty model with the 11 sensor-derived features, as shown in Figure 2. Therefore, we suggest that the four optimal features are sufficient to identify physical frailty and frailty phenotypes (slowness, weakness, exhaustion, and inactivity), which can reduce the complexity when analyzing and interpreting sensor-derived features.

Wearable sensor technologies for monitoring daily physical activity and health-related signals (e.g., heart rate, calorie burn, and blood oxygen level) continue to evolve, and their use is widespread (see [41,42] for review). Clearly, wearable sensor technologies have been accepted by the health care industry [43]. Therefore, objective assessment of physical frailty using wearable sensors can provide a means for the routine screening and monitoring of physical frailty irrespective of setting, which can eventually improve clinical assessments and interventions. Moreover, the expanded deployment of wearable sensors can reduce the costs of health care by reducing the number of patient visits and the costs of analysis. Taken together, we suggest that our results will improve the triaging/screening and monitoring of physical frailty remotely and in clinical settings, facilitate the tracking of changes in physical frailty and frailty phenotypes over time, and complement the use of two common physical frailty assessment methods that convey other types of health information.

Our study is limited by the possibility that our logistic regression model with optimal sensor-derived features may misidentify robust individuals as having physical frailty based on a sensitivity of 72.2%, specificity of 70.0%, and accuracy of 71.3%. We attribute the possible misidentifications to the differences between the Fried frailty phenotype and the sensor-derived features, and the binary allocation of the Fried frailty phenotypes. The sample size (RG (*n* = 73) and FG (*n* = 186)) and gender imbalance may be insufficient even though we used the bootstrapping technique with subsampling to generalize logistic regression modeling.

## 5. Conclusions

This study used machine learning combined with logistic regression modeling to identify the optimal spontaneous daily physical activity features measurable using a pendant sensor required for identifying physical frailty and frailty phenotypes (slowness, weakness, exhaustion, and inactivity). Of the 12 features derived from pendant sensor data, the four optimal features were % of standing, % of walking, walking cadence, and longest walking bout. Walking cadence is an indicator of slowness/weakness, longest walking bout is an indicator of exhaustion, and % of standing and % of walking are indicators of inactivity.

Our findings should inform the future design and implementation of wearable sensor technologies as a remote patient monitoring tool for the routine screening and monitoring of physical frailty and frailty phenotypes. Future research will focus on improving identification rates by using a multiclass classification method and larger samples with balanced gender within and between groups. We will also study the use of wearable sensor technologies integrated with smartphone apps for the remote screening and monitoring of older adults with, or at risk of, physical frailty.

## Figures and Tables

**Table 1:** Description of 12 sensor-derived features and associated phenotypes.

Sensor-Derived Feature	Description	Phenotype
Walking cadence	Number of steps per minute in walking, 90th percentile	Slowness/weakness
Number of stand-to-sit	Postural transitions from a standing position to a sitting position	Slowness/weakness
Duration of stand-to-sit	Duration of stand-to-sit transitions, 90th percentile	Slowness/weakness
Number of sit-to-stand	Postural transitions from a sitting position to a standing position	Slowness/weakness
Duration of sit-to-stand	Duration of sit-to-stand transitions, 90th percentile	Slowness/weakness
Longest walking bout	Number of steps for longest unbroken walking	Exhaustion
Walking steps per episode	Average of number of steps per unbroken walking	Exhaustion
Walking steps	Number of total walking steps	Inactivity
% of sitting	Percentage of sitting time for 24 h	Inactivity
% of standing	Percentage of standing time for 24 h	Inactivity
% of walking	Percentage of walking time for 24 h	Inactivity
% of lying	Percentage of lying time for 24 h	Inactivity

**Table 2:** Demographics and frailty phenotypes for robust group (RG) and pre-frail/frail group (FG).

	No./Total No. (%) by Group		*p*-Value
	RG (*n* = 73)	FG (*n* = 186)	
Age, years	74.4 ± 6.6	76.6 ± 8.4	0.092
Female, *n* (%)	53 (72.6)	115 (61.8)	0.102
Height, cm	165.1 ± 8.9	163.3 ± 10.1	0.185
Weight, kg	74.8 ± 19.0	79.2 ± 17.9	0.056
BMI, kg/m^2^	24.6 ± 8.0	29.1 ± 8.0	<0.0001 *
Frailty phenotype, 0–5	0	1.5 ± 1.3	–
Presence of slowness, *n* (%)	0 (0)	129 (69.4)	–
Presence of weakness, *n* (%)	0 (0)	119 (64.0)	–
Presence of exhaustion, *n* (%)	0 (0)	56 (30.1)	–
Presence of weight loss, *n* (%)	0 (0)	17 (9.1)	–
Presence of inactivity, *n* (%)	0 (0)	75 (40.3)	–

Values are presented as mean ± standard deviation or *n* (%). Asterisk denotes a significant difference between the groups.

**Table 3:** Comparison of sensor-derived features between the robust group (FG) and pre-frail/frail group (FG).

Sensor-Derived Feature	Unit	RG (*n* = 73)	FG (*n* = 186)	*p*-Value	Effect Size
Walking cadence	steps/min	115.3 ± 9.0	108.8 ± 16.2	<0.0001 *	0.50
Number of stand-to-sit	*n*	134.2 ± 70.8	114.3 ± 59.9	0.032 *	0.30
Duration of stand-to-sit	s	3.9 ± 0.6	4.3 ± 1.2	0.004 *	0.42
Number of sit-to-stand	*n*	137.0 ± 66.0	117.1 ± 59.0	0.023 *	0.32
Duration of sit-to-stand	s	3.9 ± 0.6	4.6 ± 1.5	<0.0001 *	0.61
Longest walking bout	*n*	1372.7 ± 1702.5	472.9 ± 749.8	<0.0001 *	0.68
Walking steps per episode	*n*	32.5 ± 18.9	25.4 ± 13.8	0.003 *	0.43
Walking steps	*n*	4788.5 ± 2667.6	3004.6 ± 2531.5	<0.0001 *	0.69
% of sitting	%	34.3 ± 11.0	37.6 ± 13.9	0.040 *	0.26
% of standing	%	17.9 ± 4.8	13.7 ±5.5	<0.0001 *	0.81
% of walking	%	6.8 ± 3.1	4.3 ± 3.0	<0.0001 *	0.82
% of lying	%	41.0 ± 12.3	44.4 ± 16.4	0.117	0.24

Values are presented as mean ± standard deviation. Asterisks denote the significant difference between groups.

**Table 4:** Results of area-under-curve (AUC) for the physical frailty, slowness, weakness, exhaustion, and inactivity models.

Model (Dependent Variable)	Features (Independent Variables)	AUC
Physical frailty	Walking cadence, Number of stand-to-sit, Duration of stand-to-sit, Number of sit-to-stand, Duration of sit-to-stand, Longest walking bout, Walking steps per episode, Walking steps, % of sitting, % of standing, % of walking	0.80
Slowness	Walking cadence	0.74
	Number of stand-to-sit	
	Duration of stand-to-sit	
	Number of sit-to-stand	
	Duration of sit-to-stand	
Weakness	Walking cadence	0.71
	Number of stand-to-sit	
	Duration of stand-to-sit	
	Number of sit-to-stand	
	Duration of sit-to-stand	
Exhaustion	Longest walking bout	0.60
	Walking steps per episode	
Inactivity	Walking steps	0.72
	% of sitting	
	% of standing	
	% of walking	

**Table 5:** Ranking of 11 sensor-driven features identified from machine learning.

Rank	Sensor-Derived Features	Phenotype
1	% of standing	Inactivity
2	% of walking	Inactivity
3	Walking cadence	Slowness/weakness
4	Longest walking bout	Exhaustion
5	Walking steps per episode	Exhaustion
6	% of sitting	Inactivity
7	Duration of sit-to-stand	Slowness/weakness
8	Walking steps	Inactivity
9	Duration of sit-to-stand	Slowness/weakness
10	Number of stand-to-sit	Slowness/weakness
11	Duration of stand-to-sit	Slowness/weakness

**Table 6:** Model validation.

Validation Metric	Mean	95% Confidence Interval
AUC (%)	75.4	75.3 to 75.5
Sensitivity (%)	70.5	70.1 to 70.8
Specificity (%)	69.0	68.7 to 69.3
Accuracy (%)	69.5	69.4 to 69.7
PPV (%)	69.6	69.4 to 69.9
NPV (%)	70.1	69.9 to 70.4

AUC: area under the receiver operating characteristic curve. PPV: positive predictive value. NPV: negative predictive value.

## Data Availability

The datasets are available upon request to the corresponding author.
